# Editorial: Frontiers in oral health: Highlights in preventive dentistry 2021/2

**DOI:** 10.3389/froh.2022.1105724

**Published:** 2023-01-10

**Authors:** Yasmi O. Crystal, Guglielmo Campus, Joana Cunha-Cruz

**Affiliations:** ^1^ Department of Pediatric Dentistry, New York University College of Dentistry, New York, NY, United States; ^2^ Department of Pediatric and Preventive Dentistry, University of Bern, Bern, Switzerland; ^3^ Department of Clinical and Community Sciences, School of Dentistry, University of Alabama at Birmingham, Birmingham, AL, United States

**Keywords:** prevention, advocacy, public policy, oral health, COVID-19

**Editorial on the Research Topic** Frontiers in oral health: Highlights in preventive dentistry 2021/2

Prevention is a broad and challenging term. When one hears the word, first thoughts are usually directed to the interventions that could be implemented to prevent a disease from occurring in an individual or a population (primary prevention). One could also think of early screening to identify and reverse the progress of diseases in their earliest stages (secondary prevention), or of ways to effectively manage a disease post diagnosis to slow down or stop its progression (tertiary prevention) (1).

Research on specific therapies and techniques that can foster prevention at any level, and its dissemination in scientific journals and the public press, are imperative to establish a solid basis for the implementation of oral health prevention practices and policies worldwide. However, new preventive drugs and technology alone cannot help us solve the long-standing problems that we face with oral diseases, unless a roadmap for implementation has been secured through an effective prevention framework that is well understood and established for each specific system/region. Governments, policy makers, insurers, organized dentistry, industry, oral health care providers at all levels, the institutions that educate them, and individual patients all have a role to play in the carefully orchestrated development of a care model of health promotion and prevention (2).

The Research Topic *Frontiers in Oral Health: Highlights in Preventive Dentistry 2021/2* includes a series of eight articles, each individually addressing one of the many pieces of the puzzle that must be put into place to lead to the effective prevention of oral diseases, including special considerations as we continue dealing with the COVID-19 pandemic.

Prevention strategies at the patient level are presented by two reviews: one on effectiveness of chewing gum in reducing plaque levels (Nasseripour et al.) and another one on impact on quality of life of dry mouth in Sjögren's Syndrome (Ngo and Thompson). The dysbiosis of oral microorganisms in plaque is a determinant risk factor for the development of oral diseases like caries and periodontal disease. Interventions to reduce dental plaque include chewing sugar-free gum (SFG) as an adjunct to toothbrushing. The systematic review and meta-analysis conducted by Nasseripour et al. explores the role of sugar-free chewing gum on plaque quantity in the oral cavity, by looking at the differences in the levels of plaque quantity in adults and children who chew SFG, compared with those who do not chew SFG, who do not chew gum, or who use alternatives such as probiotics or fluoride varnish. The authors conclude that there is some evidence that chewing SFG, in particular xylitol SFG, reduces the quantity of plaque in the oral cavity in comparison to non SFG chewing or no chewing controls while calling for further research on the topic.

Another important risk factor for the development of diseases like dental caries and periodontitis, is dry mouth, which is a main symptom of patients with Sjögren's Syndrome (SS). The mini review by Ngo and Thomson provides an update on the impact of this condition on the quality of life of persons with SS. Understanding of this topic from a patient-centered point of view is crucial for the choice and implementation of preventive measures and disease impact interventions that can aid patients with SS and others with dry mouth.

The article by Alai-Towfigh et al. shifts to a provider-centered perspective, reporting the findings of a survey to assess Canadian dentists' views on the first dental visit for children. A first dental visit by 12 months of age is the official position of many dental organizations, with the aim of identifying children at high-risk to develop Early Childhood Caries and to establish the appropriate preventive measures. Their finding that the majority of Canadian general dentists do not follow this recommendation is followed by a discussion of the reasons why, and analysis of how provider characteristics influence their adherence to the recommendation. These results will be the basis for targeted educational campaigns for dentists to increase participation in early visits, as they are instrumental for the prevention of caries in children.

Two additional articles in this Research Topic address the importance of dental education in the career paths of future dentists. In their review, Jiang et al. report that fresh dental graduates in Japan, Hong Kong, and Mainland China are unfamiliar with potential career paths. They discuss the possibilities in the many different options that are available to dentists in these regions, and stress that alongside providing professional training to dental students, it is very important for educators to guide students to explore different career paths.

Additionally, the research report by Amini et al. explores how oral health advocacy education impacts future engagement. Advocacy for oral health is crucial for advancing oral health-related policies to decrease the prevalence and burden of oral diseases. However, there is limited exposure to legislative advocacy in predoctoral dental education. Their results point out that dental students with advocacy experience are more likely to report intentions to participate in advocacy as dentists, and they conclude that dental education has a critical role in preparing future dentist-advocates. Effective advocacy to influence government agencies to incorporate prevention into dental benefit funding for all ages is critical worldwide to change the behavior of dentists from a traditional surgical/operative approach to prevention and optimal intervention.

The restrictions that the COVID-19 pandemic has placed on the delivery of traditional preventive procedures worldwide, added to all the measures that we have had to establish to prevent the widespread of the virus within the healthcare personnel and in the population, have been an extreme challenge. The last three articles explore how the COVID-19 pandemic has affected the provision of dental care.

Jiang et al. describe the changes in oral health policies and guidelines in response to the pandemic in 9 countries. The commentary by Singhal et al. expands on the policies that took place in additional provinces and territorial jurisdictions in Canada throughout the pandemic. Finally, the research article by Sofi-Mahmudi and Raittio assesses the adherence to transparency practices of shared data from open access COVID-19 related articles published in dental journals indexed in PubMed. The authors conclude that while the majority of the papers disclosed conflicts of interest, the prevalence of other transparency practices was far from the acceptable level, and a much stronger commitment to open science practices, particularly to preregistration and data and code sharing, is needed from all stakeholders.

In conclusion, this Research Topic includes original research and review papers highlighting the many roles of different stakeholders for advancing the science of the prevention of oral diseases in different regions of the world. Dentists, oral health care providers at all levels and settings, dental educators, third party payers, researchers, and policy makers should find this information useful to establish collaborations that lead to the prevention of disease and maintenance of sustained health of the populations they serve.
